# High PPP4C expression predicts poor prognosis in diffuse large B-cell lymphoma

**DOI:** 10.1007/s10238-024-01356-6

**Published:** 2024-04-29

**Authors:** Xue Hui, Liru Li, Wenjing Xiong, Yue Liu, Hongbin Li, Han Zhang, Shu Zhao, Yue Zhang

**Affiliations:** Department of Clinical Oncology, Harbin Medical University Cancer Hospital, 150 Haping Road, Harbin, 150040 China

**Keywords:** PPP4C, Diffuse large B-cell lymphoma, Prognosis biomarker, Bioinformatic analysis

## Abstract

The significance of Protein phosphatase 4 catalytic subunit (PPP4C) in diffuse large B-cell lymphoma (DLBCL) prognosis is not well understood. This work aimed to investigate the expression of PPP4C in DLBCL, investigate the correlation between PPP4C expression and clinicopathological parameters, and assess the prognostic significance of PPP4C. The mRNA expression of PPP4C was investigated using data from TCGA and GEO. To further analyze PPP4C expression, immunohistochemistry was performed on tissue microarray samples. Correlation analysis between clinicopathological parameters and PPP4C expression was conducted using Pearson's chi-square test or Fisher's exact test. Univariate and multivariate Cox hazard models were utilized to determine the prognostic significance of clinicopathological features and PPP4C expression. Additionally, survival analysis was performed using Kaplan–Meier survival curves. In both TCGA and GEO datasets, we identified higher mRNA levels of PPP4C in tumor tissues compared to normal tissues. Upon analysis of various clinicopathological features of DLBCL, we observed a correlation between high PPP4C expression and ECOG score (*P* = 0.003). Furthermore, according to a Kaplan–Meier survival analysis, patients with DLBCL who exhibit high levels of PPP4C had worse overall survival (*P* = 0.001) and progression-free survival (*P* = 0.002). PPP4C was shown to be an independent predictive factor for OS and PFS in DLBCL by univariate and multivariate analysis (*P* = 0.011 and* P* = 0.040). This study's findings indicate that high expression of PPP4C is linked to a poor prognosis for DLBCL and may function as an independent prognostic factors.

## Introduction

Diffuse large B-cell lymphoma (DLBCL) is the most common aggressive lymphoma, accounting for 30–40% of all non-Hodgkin's lymphoma (NHL) [1]. It displays a great deal of heterogeneity in terms of clinical symptoms, pathological features, responsiveness to treatment, and prognostic variability [1, 2]. The prognosis for DLBCL has significantly improved recently with the use of rituximab targeted therapy [3]. However, about 30% of DLBCL patients relapse after receiving standard therapy or become insensitive to the first course of treatment [4–6]. Therefore, it is crucial for the therapy of DLBCL to identify novel biomarkers with malignant function as well as prognostic significance.

Phosphorylation is an important component of the post-translational modifications of proteins. About 30% of proteins in human cells are modified by phosphorylation, and this phosphorylation is one of the important mechanisms regulating cell proliferation, differentiation, and other cellular functions [7, 8]. Previously, protein phosphatases have been considered as silent chaperones accompanying protein kinases, randomly reversing the phosphorylation of protein kinases, but increasing evidence has shown that protein phosphatases constitute a complex and diverse family of enzymes parallel to protein kinases, which can actively and specifically dephosphorylate specific substrates, and are closely related to the development of malignancy.

Currently identified that 107 subfamilies of protein phosphatases, which can be divided into the following four categories: phosphoprotein phosphatases (PPPs), protein tyrosine phosphatases (PTPs), metal-dependent protein phosphatases (PPMs) and phosphatases of the haloacid dehalogenases (HAD phosphatase). Among them, PPPs, as the most diverse and the most classic family, can be divided into PP1, PP2A, PP4, PP5, PP6 and PP7 [9].

PP4, independent of other phosphatases, is involved in a variety of pathophysiological processes, and has attracted increasing attention from many researchers. PP4 is structurally similar to PP2A in that it exists and functions as a heteromultimer in the form of the catalytic subunit PPP4C and different regulatory subunits [10]. PPP4C, the core catalytic subunit of PP4, is localized in the 16q11.2 region of human chromosome and was initially isolated and identified from rabbit liver cDNA library by Brewis ND et al. [11, 12]. With the further study on the function of PPP4C gene, it has been found that PPP4C is involved in several pathophysiological processes such as microtubule formation [13], spliceosomal complex assembly [14], immune regulation, DNA damage repair [15–18], and can promote cell proliferation, inhibit cell apoptosis, regulate NF-κB, JNK, mTOR and other malignancy related signaling pathways [19, 20]. Relevant studies have found that the abnormal expression of the PPP4C gene exists in solid tumors such as lung cancer [21], breast cancer [21], pancreatic ductal adenocarcinoma [22], glioma cancer [23], and colorectal carcinoma [24], and affects the biological characteristics of tumor proliferation, migration, and invasion. However, there have been no reports of PPP4C in DLBCL. The role of PPP4C in DLBCL, including its prognostic significance, remains to be further determined.

Therefore, in this study, we initially conducted a comparative analysis of the mRNA expression levels of PPP4C in DLBCL and non-tumoral tissues. Subsequently, we used immunohistochemistry to detect the expression profile of PPP4C in DLBCL tissue chips. This allowed us to investigate the correlation between the expression level of PPP4C and the clinical characteristics and survival outcomes of DLBCL patients. The ultimate goal was to elucidate the clinical significance of PPP4C in DLBCL patients.

## Materials and methods

### PPP4C gene expression analysis

Based on TCGA and GTEx database, PPP4C expression in normal and tumor tissues of 34 types of human cancer was investigatede (https://portal.gdc.cancer.gov/). In DLBCL, 447 normal tissues and 47 tumor tissues were compared. Besides, the GEO datasets GSE56315, GSE10846 and GSE32018 provided us with the gene expression profiles to explore PPP4C expression (http://ncbi.nlm.nih.gov/geo/).

### Tissue microarray

A tissue microarray including 190 human DLBCL sample tissues that were not treated was obtained. The samples were taken at the Harbin Medical University Cancer Hospital in China between November 2008 and April 2018. The diagnosis and classification of DLBCL is determined by the WHO classification method and all cases have received a standardized treatment programme of CHOP/CHOPE or R-CHOP/CHOPE for at least 4 cycles. Clinical data, including gender, age, B symptoms, LDH level, gene expression profiles, ECOG score, Ann Arbor stage, extranodal invasion, IPI score, Hans typing, KI67 expression profiles, therapeutic regimens, and survival status, were collected from medical records. The work was reviewed and approved by the Harbin Medical University Ethical Committee, and written informed consent was obtained from each participant. The patient's clinical characteristics are listed in Table 1.

**Table 1:** Clinical data of 190 cases of DLBCL

Clinical characteristics	Total patients (*n*, %)
Gender	
Female	100(52.6)
Male	90(47.4)
Age	
≤ 60	128(67.4)
> 60	62(32.6)
Ann Arbor stage	
I + II	106(57.7)
III + IV	84(44.3)
ECOG	
0–1	123(64.7)
≥ 2	67(35.3)
Extranodal invasion	
0–1	157(82.6)
≥ 2	33(17.4)
LDH (U/L)	
Normal	131(68.9)
High	59(31.1)
IPI	
≤ 2	137(72.2)
> 2	53(27.8)
B symptoms	
No	148(77.8)
Yes	42(22.2)
Hans typing	
GCB	81(42.6)
Non-GCB	109(57.4)
Ki-67	
≤ 70	85(44.7)
> 70	105(55.3)

### Follow-up

The clinical and pathological records of all patients in this study were regularly reviewed. Survival of the patients was measured using overall survival (OS), which defines the time from the date of diagnosis until the date of death from all causes or the final follow-up. Progression-free survival (PFS), on the other hand, referred to the period from the date of diagnosis to the date of disease progression, relapse, or death. Data for this study were obtained from clinical records or telephone interviews with the patients or their relatives, and the last date of follow-up was January 1, 2022. This approach ensured comprehensive and accurate information regarding the patients' outcomes throughout the study period.

### Immunohistochemistry

The tissue sections embedded in paraffin were eliminated in dimethyl benzene and rehydrated using ethanol solutions of varying concentrations. Antigen retrieval was carried out by high-pressure repair in sodium citrate (pH 6.0) at 0.06–0.12 MPa for three minutes after deparaffinization and hydration, and then cooling to room temperature. Following a 5-min wash in phosphate-buffered saline (PBS), the sections that had been cleaned were exposed to 3% H_2_O_2_ for ten minutes at room temperature. After an overnight incubation at 4 °C with the primary antibody PPP4C (1:100; Affinity Biosciences), the tissue slices were treated in the dark with a universal anti-rabbit secondary antibody (ZSGB-BIO; China) tagged with HRP. The immunostaining was visualized with diaminobenzidine (DAB), followed by counterstaining the tissue sections with hematoxylin, dehydrating, and mounting them.

### Immunohistochemical scoring

Two independent pathologists, blinded to clinicopathological information, assessed the level of PPP4C expression based on the percentages of positive cells and staining intensity. The IHC scoring system used in this study included a grading system for the proportion of positive cells (0–5% graded as 0, 6–25% graded as 1, 26–50% graded as 2, 51–75% graded as 3, and 76–100% graded as 4) and for staining intensity (graded as 1 for weak, 2 for moderate, and 3 for strong). Consequently, the IHC scores for PPP4C expression levels were calculated by multiplying the proportion of positive cells by the staining intensity. The final definition of the PPP4C expression was as follows: 0 points for negative ( −); 1–4 points for low expression; 5–8 points for moderate (+ +); and 9–12 points for high expression (+ + +). Based on a PPP4C median IHC score of 4, all DLBCL patients were split into two groups: low and high PPP4C expression for future research.

### Statistical analysis

All statistics were performed by SPSS 18.0 statistical software and GraphPad Prism. The connection between PPP4C expression and clinicopathological parameters was examined using Pearson's chi-square test or Fisher's exact test. Cox univariate and multivariate regression models were used to evaluate the impact of different variables on Survival. For survival analysis, the Kaplan–Meier survival curve was employed. Statistical significance was defined as *P* < 0.05.

## Results

### mRNA expression level of PPP4C in DLBCL

**Fig. 1:**
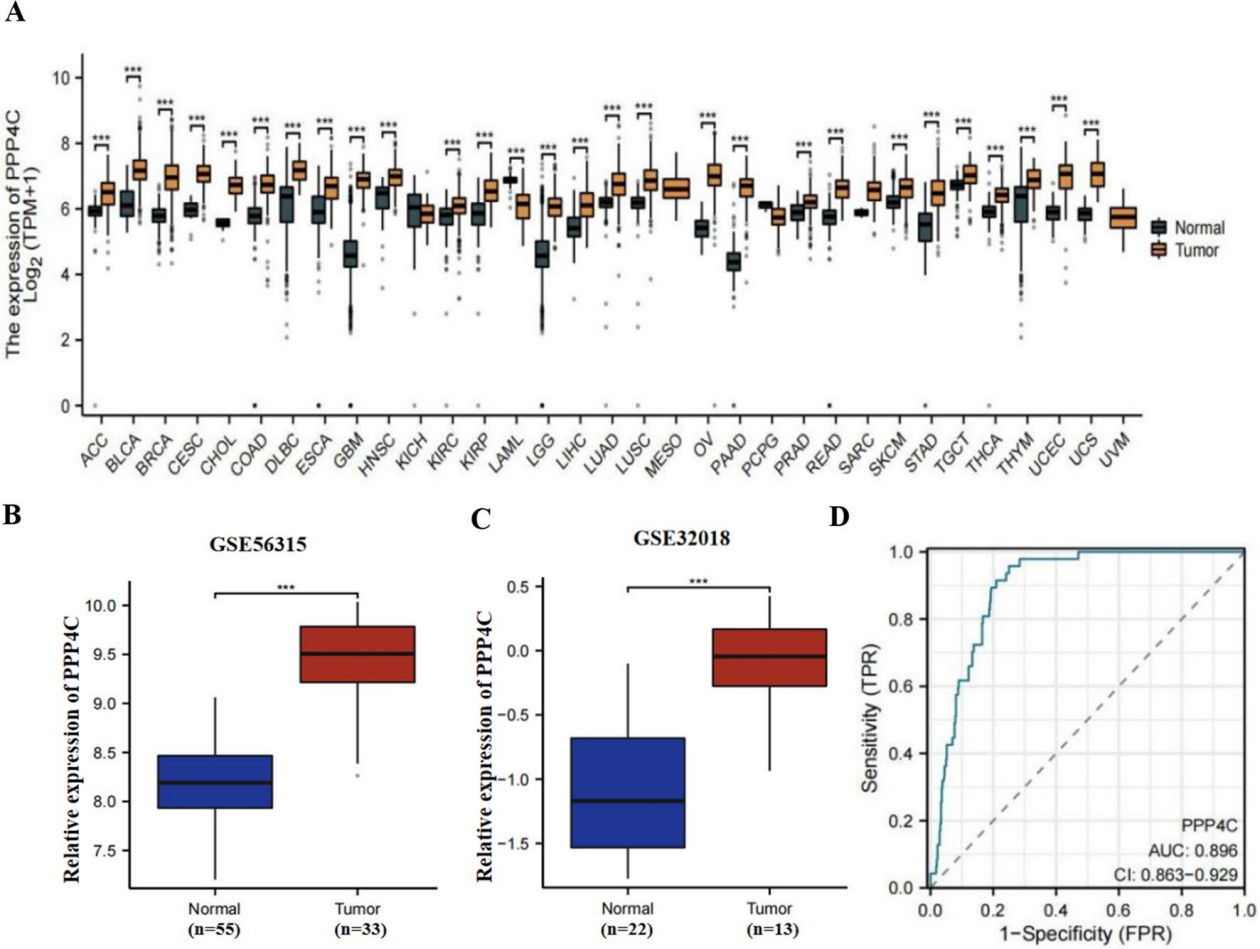
The expression profile of PPP4C in diffuse large B-cell lymphoma. **A** PPP4C expression in 34 kinds of normal and cancerous tissues (TCGA and GTEx normal data in comparison with TCGA cancer data). **B**–**C** In the GSE56315 and GSE32018 datasets, the expression level of PPP4C was greater in DLBCL tissue compared to the nearby normal tissue. **D** PPP4C demonstrated good accuracy in predicting both normal and malignant outcomes, according to the ROC curve.(*p ≤ 0.05, **p ≤ 0.01,***p ≤ 0.001)

First, we utilized data from the TCGA database to analyze the gene expression levels of PPP4C in various human cancer tissues and compared them with normal tissues. Our analysis revealed that the mRNA expression of PPP4C was notably elevated in multiple types of cancer tissues, including DLBCL, in comparison to their corresponding normal tissues (Fig. 1a). Further validation of the PPP4C expression level was conducted using two separate GEO datasets: GSE56315 and GSE32018. In both datasets, PPP4C expression was greater in DLBCL tumor tissues rather than normal tissues (Fig. 1b and c). PPP4C has good sensitivity and specificity for predicting patient outcomes, according to ROC analysis (AUC 0.896; Fig. 1d).

### PPP4C expression levels in tissue samples of DLBCL patients

**Fig. 2:**
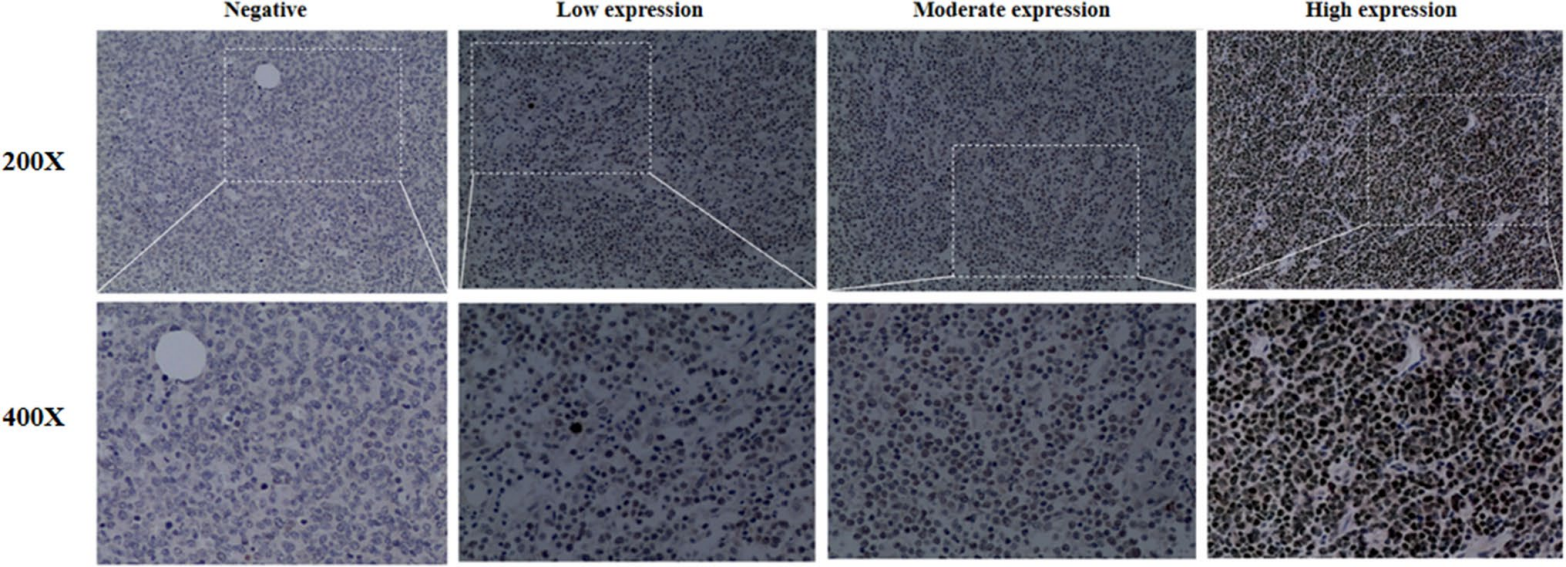
Typical IHC pictures of the expression of PPP4C in tissues from DLBCL patients

Next, we conducted an immunohistochemical assay to assess the expression of PPP4C in the DLBCL tissue microarray. The findings revealed that 76.84% (146/190) of DLBCL tissues exhibited positive staining of PPP4C, primarily localized in the nucleus of tumor cells. Of these, 128 patient samples (or 67.4%) were classified as having PPP4C low expression, and the remaining 62 samples (or 32.6%) were classified as having high PPP4C expression. Figure 2 displays various PPP4C IHC staining intensity.

### Correlation between PPP4C expression and clinical characteristics of DLBCL patients

We conducted an additional analysis to examine the relationship between PPP4C expression levels and clinicopathological parameters in 190 DLBCL patients. Using a median IHC score of 4 as the cut-off value for PPP4C expression, we found high expression in 62 (32.63%) tissue samples from DLBCL patients. Next, the association of PPP4C expression in tumor tissues was evaluated with various clinicopathologic parameters including sex, age of diagnosis, Ann Arbor stage, ECOG, extranodal site, LDH, IPI score, B symptoms, Hans typing, and Ki-67. Table 2 summarizes the correlation between PPP4C expression and clinicopathological parameters in 190 DLBCL patients. High PPP4C expression was found to be significantly associated with higher ECOG (*P* = 0.003) scores compared to low PPP4C expression. However, no significant correlation was observed between PPP4C expression level and other clinicopathological parameters.

**Table 2:** Relationship between PPP4C expression and clinicopathologic parameters

Clinical characteristics	Total patients (*n*, %)	PPP4C		χ2	*P*
		Low expression	High expression		
Gender					
Female	100(52.6)	66	34	0.180	0.672
Male	90(47.4)	62	28		
Age					
≤ 60	128(67.4)	88	40	0.341	0.559
> 60	62(32.6)	40	22		
Ann Arbor stage					
I + II	106(57.7)	76	30	2.045	0.153
III + IV	84(44.3)	52	32		
ECOG					
0–1	123(64.7)	92	31	8.755	0.003
≥ 2	67(35.3)	36	31		
Extranodal invasion					
0–1	157(82.6)	107	50	0.253	0.615
≥ 2	33(17.4)	21	12		
LDH(U/L)					
Normal	131(68.9)	87	44	0.175	0.675
High	59(31.1)	41	18		
IPI					
≤ 2	137(72.2)	98	39	–	0.058
> 2	53(27.8)	30	23		
B symptoms					
No	148(77.8)	100	48	0.012	0.912
Yes	42(22.2)	28	14		
Hans typing					
GCB	81(42.6)	51	30	1.247	0264
Non-GCB	109(57.4)	77	32		
Ki-67					
≤ 70	85(44.7)	56	29	0.155	0.694
> 70	105(55.3)	72	33		

### Prognostic value of PPP4C in DLBCL

To assess whether PPP4C expression and clinicopathological characteristics are independent risk factors for DLBCL patients, univariate and multivariate Cox regression analyses were conducted. The univariate analysis revealed significant associations between decreased overall survival (OS) and several factors, including stage III/IV (*P* < 0.001), ECOG ≥ 2 score (P = 0.038), above normal LDH levels (*P* = 0.013), IPI > 2 score (*P* < 0.001), absence of rituximab use (*P* = 0.021), and high PPP4C expression levels (*P* = 0.001) (OS; Table 3).Similarly, stage III/IV (*P* < 0.001), above normal LDH levels (*P* = 0.018), Extranodal site ≥ 2 (*P* = 0.035), IPI > 2 score (*P* < 0.001), absence of rituximab use (*P* = 0.039) and high PPP4C expression levels (*P* = 0.002) were also found to be significantly associated with decreased progression-free survival (PFS; Table 4) in the univariate analysis. Furthermore, the multivariate Cox model analysis identified IPI (*P* = 0.043), use of rituximab (*P* = 0.004), and PPP4C levels (*P* = 0.011) as independent predictors for OS, while also indicating that IPI (*P* = 0.018), use of rituximab (*P* = 0.029), and PPP4C levels (*P* = 0.040) were independent predictors of PFS.

**Table 3:** Univariate and multivariate Cox regression analysis of OS

Variable	Univariate analysis		Multivariate analysisl	
	HR (95%CI)	*P*	HR (95%CI)	*P*
Gender (Male vs. Female)	1.080 (0.671–1.738)	0.751		
Age (> 60 vs. ≤ 60)	1.553 (0.960–2.512)	0.073		
Ann Arbor stage (III–IV vs. I–II)	2.248 (1.386–3.648)	** < 0.001**	1.221 (0.573–2.603)	0.605
ECOG (≥ 2 vs. 0–1)	1.663 (1.030–2.684)	**0.038**	1.226 (0.718–2.093)	0.455
Extranodal invasion (≥ 2 vs. 0–1)	1.387 (0.770–2.498)	0.275		
LDH level (High vs. Normal)	1.848 (1.140–2.995)	**0.013**	1.406 (0.787–2.511)	0.249
IPI (3–5 vs. 0–2)	3.053 (1.890–4.933)	** < 0.001**	2.383 (1.028–5.526)	**0.043**
B symptoms (Yes vs. No)	1.055 (0.595–1.871)	0.855		
Hans typing (Non-GCB vs. GCB)	1.286 (0.786–2.106)	0.317		
PPP4C expression (High vs. Low)	2.269 (1.409–3.654)	**0.001**	1.925 (1.159–3.196)	**0.011**
Ki-67 (> 70 vs. ≤ 70)	0.737 (0.457–1.186)	0.208		
Rituximab (Yes vs. No)	0.516 (0.294–0.903)	**0.021**	0.410 (0.224–0.751)	**0.004**

*P* < 0.05 was bolded

**Table 4:** Univariate and multivariate Cox regression analysis of PFS

Variable	Univariate analysis		Multivariate analysisl	
	HR (95%CI)	*P*	HR (95%CI)	*P*
Gender (Male vs. Female)	1.179 (0.801–1.736)	0.403		
Age (> 60 vs. ≤ 60)	1.450 (0.978–2.149)	0.064		
Ann Arbor stage (III–IV vs. I–II)	2.172 (1.469–3.212)	** < 0.001**	1.445 (0.800–2.610)	0.222
ECOG (≥ 2 vs. 0–1)	1.265 (0.849–1.885)	0.248		
Extranodal invasion (≥ 2 vs. 0–1)	1.660 (1.035–2.663)	**0.035**	0.772 (0.428–1.391)	0.389
LDH level (High vs. Normal)	1.615 (1.084–2.405)	**0.018**	1.147 (0.714–1.845)	0.570
IPI (3–5 vs. 0–2)	2.780 (1.869–4.136)	** < 0.001**	2.318 (1.156–4.650)	**0.018**
B symptoms (Yes vs. No)	1.551 (0.999–2.408)	0.050		
Hans typing (Non-GCB vs. GCB)	1.328 (0.891–1.978)	0.164		
PPP4C expression (High vs. Low)	1.853 (1.253–2.739)	**0.002**	1.550 (1.020–2.356)	**0.040**
Ki-67 (> 70 vs. ≤ 70)	0.742 (0.504–1.094)	0.132		
Rituximab (Yes vs. No)	0.633 (0.410–0.977)	**0.039**	0.594 (0.372–0.948)	**0.029**

*P* < 0.05 was bolded

**Fig. 3:**
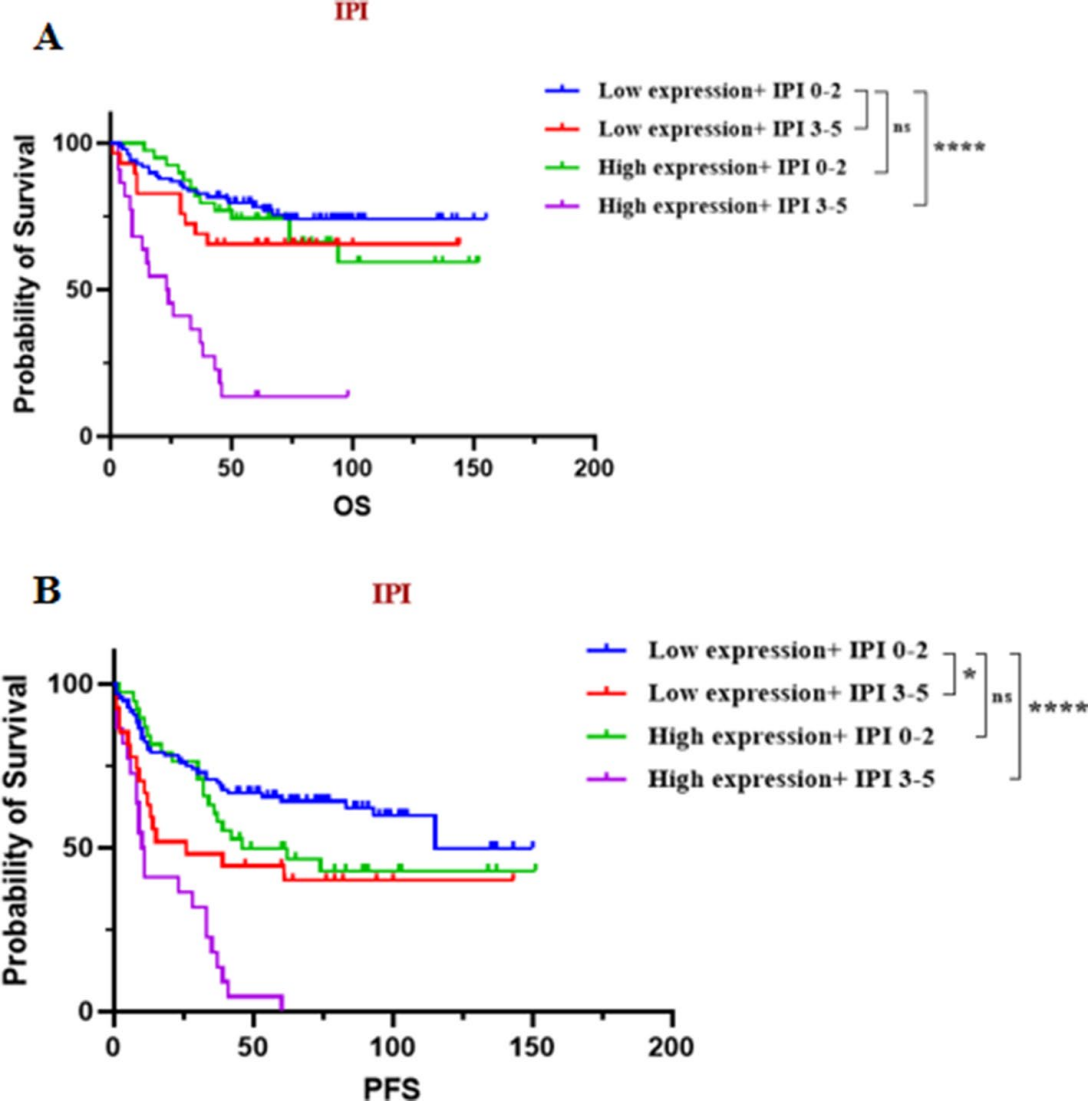
OS and PFS of DLBCL patients in different risk groups

Based on the two independent risk factors mentioned above, in order to refine the risk stratification of DLBCL, all patients were divided into four groups, and a log-rank (Mantel-Cox) test was performed to consider the interaction between PPP4C expression levels and IPI. The level of risk for poor prognosis in the remaining three groups was analyzed, using patients in the 0 risk factor group as a reference. It was found that for OS, patients in the High expression + IPI 3–5 group had a 6.2-fold higher risk of poor prognosis compared to the 0 risk factor group (HR = 6.246, 95%CI 2.425–16.09, *P* < 0.001). Similarly, for PFS, Low expression + IPI 3–5 and High expression + IPI 3–5 were 2.0 times higher (HR = 1.952, 95%CI 0.9704–3.925,* P* = 0.0217) and 4.8 times (HR = 4.753, 95%CI 2.111–10.70, *P* < 0.001). Although there was no significant correlation between the Low expression + IPI 3–5 and High expression + IPI 0–2 groups, it is still informative for refining risk stratification (Fig. 3).

### Association between PPP4C expression and survival outcome in DLBCL

**Fig. 4:**
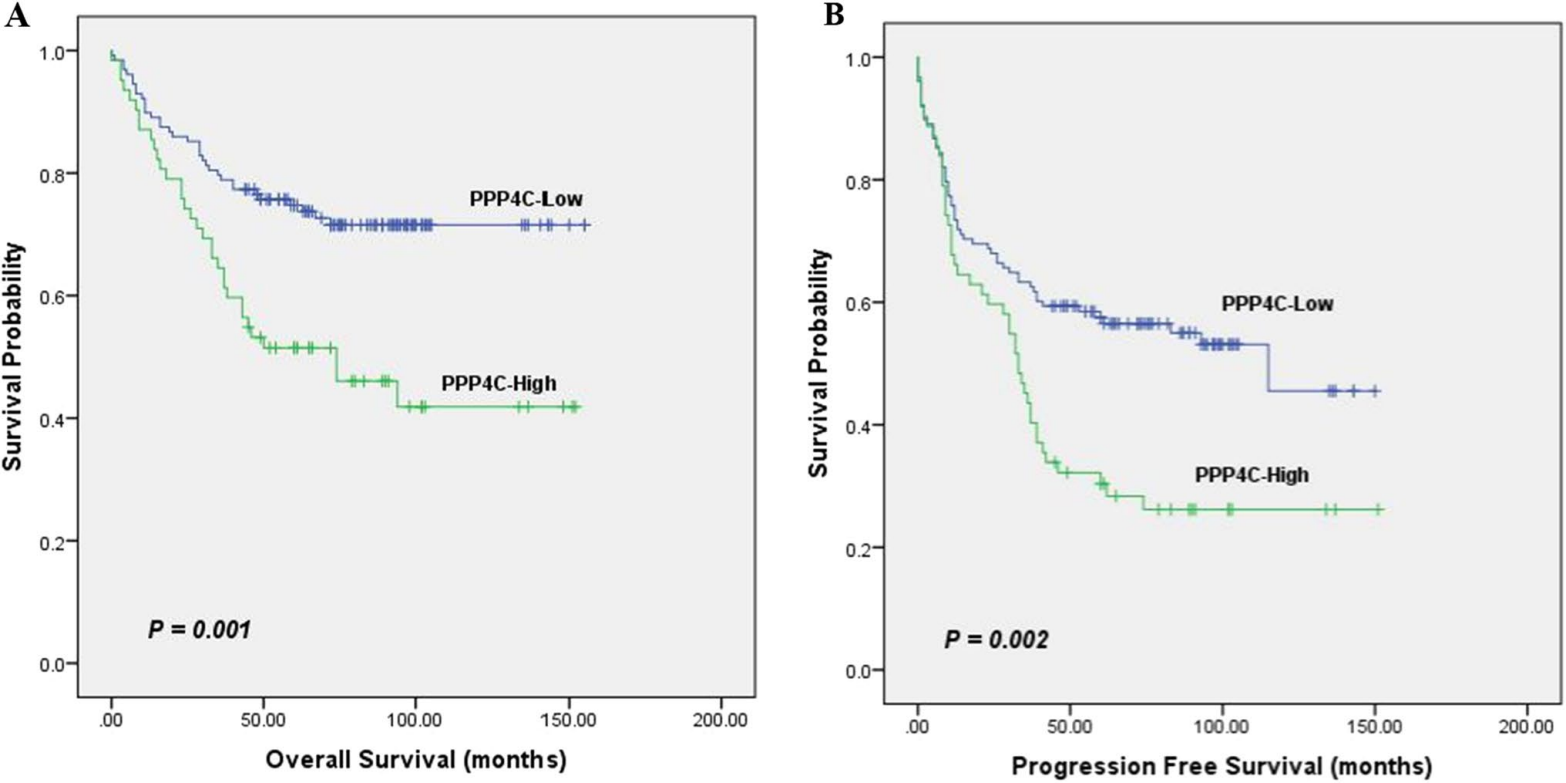
Kaplan–Meier survival curves grouped by high and low PPP4C expression in DLBCL patients

**Fig. 5:**
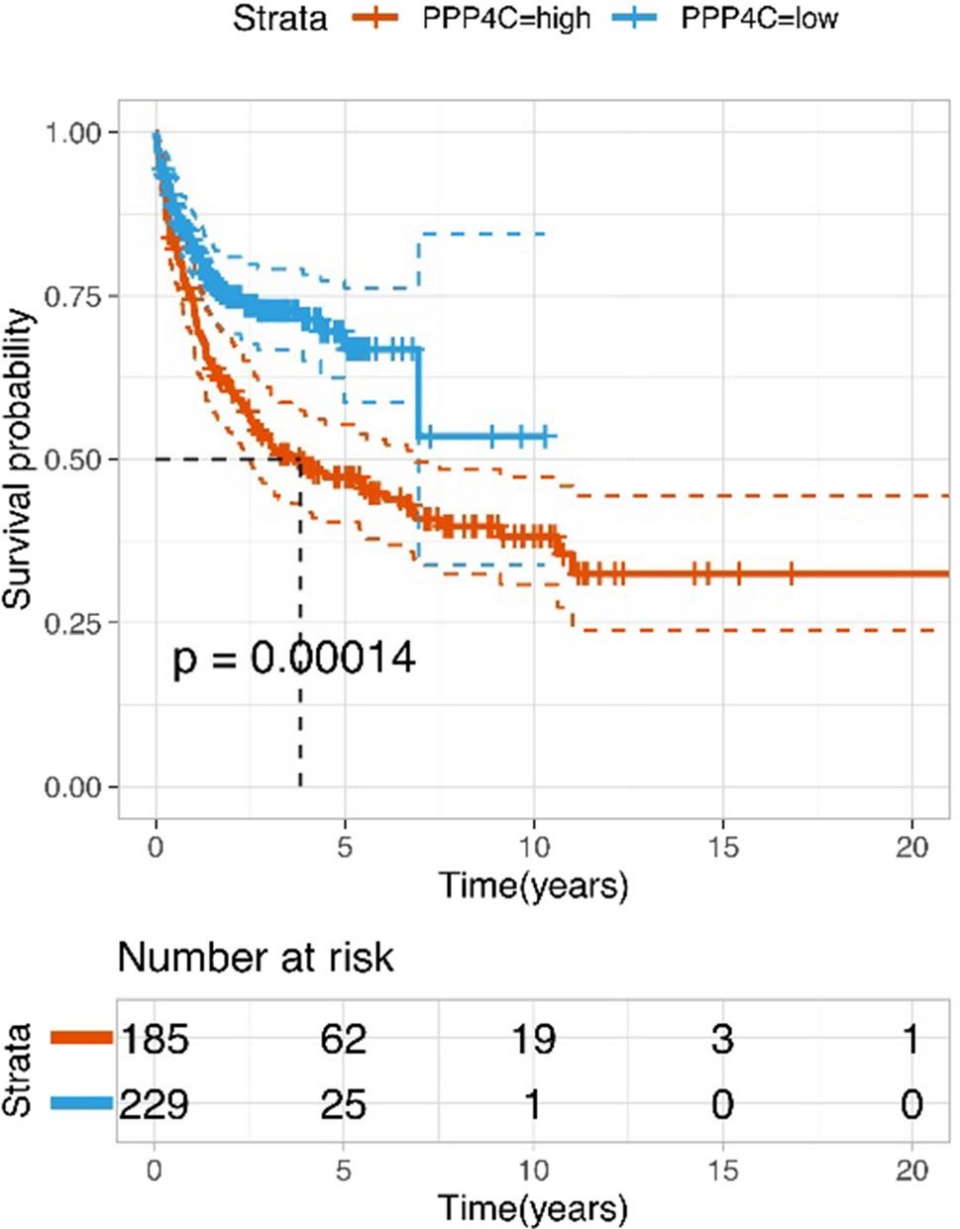
Association between PPP4C levels and survival in GSE10846

**Fig. 6:**
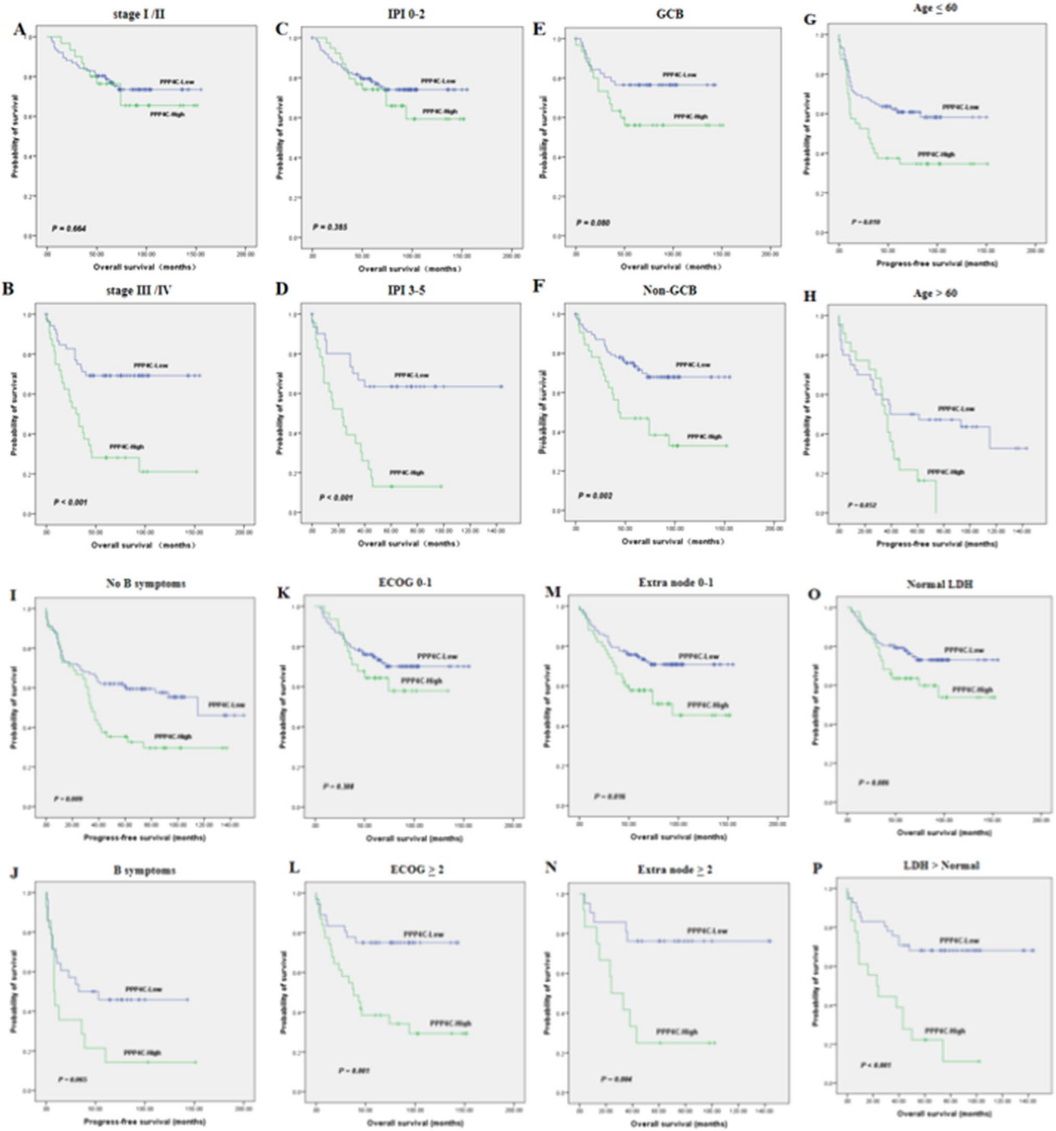
Kaplan–Meier survival curves display OS in DLBCL patients with high and low PPP4C expression, stratified by various clinical factors. **A** Stage I/II, **B** stage III/IV, **C** IPI 0–2, **D** IPI 3–5, **E** GCB, **F** non-GCB, **G** age ≤ 60, **H** age > 60, **I** No B symptoms, **J** B symptoms, **K** ECOG 0–1, **L** ECOG ≥ 2, **M** extra node 0–1, **N** extra node ≥ 2, **O** Normal LDH. **P** LDH > Normal

**Fig. 7:**
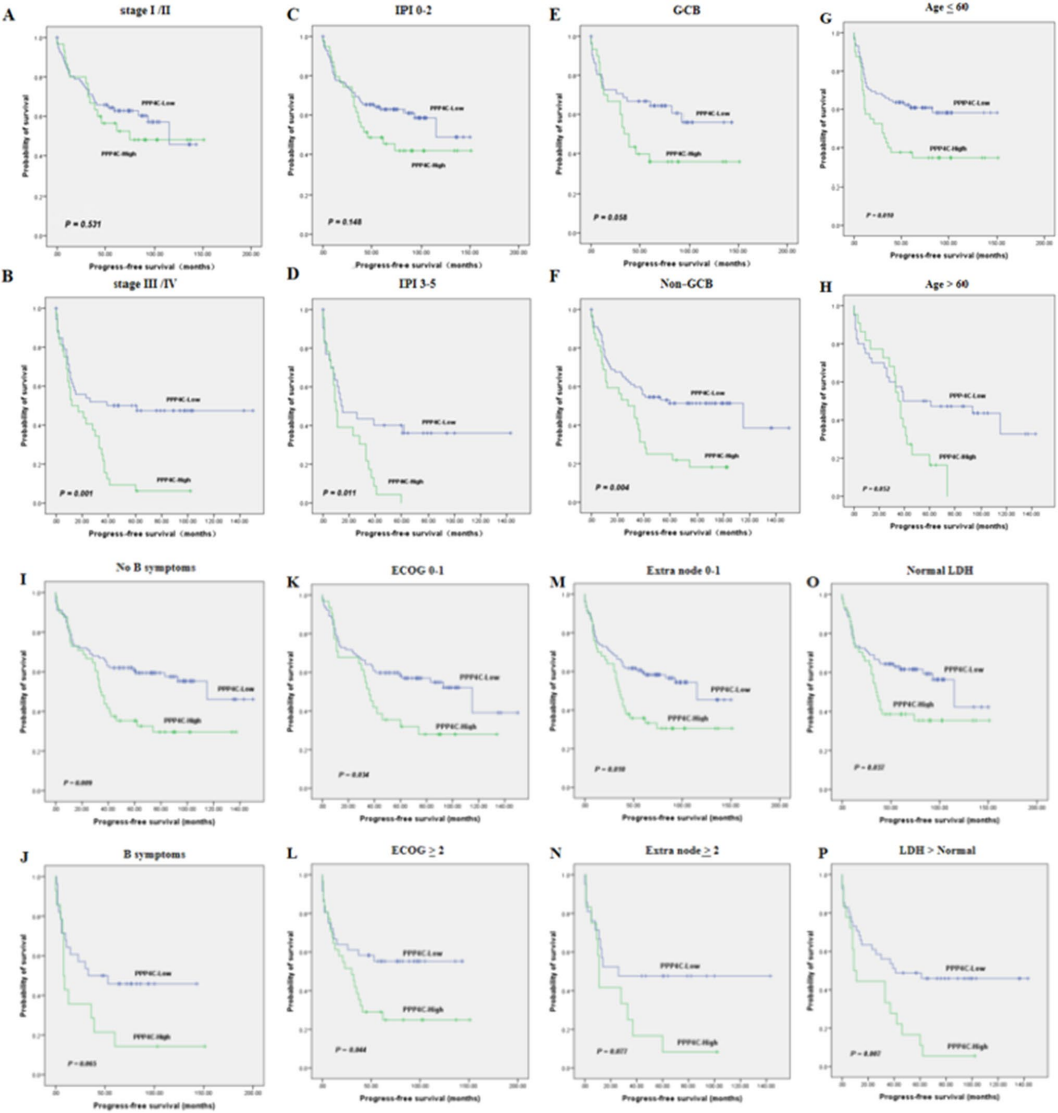
Kaplan–Meier survival curves display PFS in DLBCL patients with high and low PPP4C expression, stratified by various clinical factors. **A S**tage I/II, **B** stage III/IV, **C** IPI 0–2, **D** IPI 3–5, **E** GCB, **F** non-GCB, **G** age ≤ 60, **H** age > 60, **I** no B symptoms, **J** B symptoms, **K** ECOG 0–1, **L** ECOG ≥ 2, **M** extra node 0–1. **N e**xtra node ≥ 2, **O** Normal LDH, **P** LDH > normal

We evaluated how well PPP4C predicts OS and PFS in all cases of DLBCL. The Kaplan–Meier survival analysis showed that individuals with DLBCL who had high PPP4C expression (*n* = 62) survived considerably less than those who had low PPP4C expression (*n* = 128) (*P* = 0.001) (Fig. 4a). In the group with high PPP4C expression, 45 patients (72.6%) experienced disease progression or death, compared to 58 patients (45.3%) in the group with low PPP4C expression. PFS was statistically significant (*P* = 0.002) (Fig. 4b). We used the GEO database (*n* = 414, Fig. 5) to confirm the association between survival and PPP4C expression. Consistent with the results of this study, the results of the GEO database showed that DLBCL patients with high PPP4C expression had a worse survival prognosis. Next, we commenced our investigation by comparing the OS between patient groups with low and high PPP4C expression, diversified by different disease phenotypes (Fig. 6). Notably, our analysis revealed that in high-risk patients, PPP4C expression was substantially related to OS. Moreover, upon further analysis depicted in Fig. 7, it became evident that high PPP4C expression corresponded significantly with poorer PFS in patients of stage III/IV (*P* = 0.001), IPI 3–5 (*P* = 0.011), age ≤ 60 (*P* = 0.010), No B symptoms (*P* = 0.009) ECOG 0–1 (*P* = 0.034), ECOG ≥ 2 (*P* = 0.044), normal LDH (*P* = 0.037), LDH > normal (*P* = 0.007) and Non-GCB (*P* = 0.004) subtypes. Thus, it is observed that high PPP4C expression is linked to poorer survival outcomes in several patient subgroups.

## Discussions

DLBCL is the most common aggressive lymphoma, accounting for 30–40% of all NHLs [1]. In recent years, with the improvement of diagnosis and treatment methods, especially the advent of rituximab, the prognosis of patients has been significantly improved [3]. Even so, some patients eventually develop relapsed and refractory DLBCL, which is seriously life-threatening [4–6]. Hence, there is an immediate need to investigate efficient biomarkers for improved prognosis prediction of DLBCL patients.

Protein phosphorylation is one of the most common and important posttranslational modification forms in the human body, and as a dynamic reversible process is regulated by the competitive activity of protein kinases and protein phosphatases. Once the process is abnormal, the relevant signaling pathways will appear dysfunctional, which may lead to the occurrence of many diseases, including cancer [7, 8]. At present, the function of protein kinase has been more thoroughly studied, and has become the target of anti-tumor, but the protein phosphatase is very little studied. In recent years, with in-depth research, it has been found that protein phosphatases play an equally irreplaceable role in tumors.

PPP4C acts as the core catalytic subunit of PP4 and contains the core region of the classical catalytic subunit of the filament/threonine protein phosphatase. With the deepening of functional studies, PPP4C has been found to have its own specific regulatory subunits. At present, PP4R1, PP4R2, PP4R3, PP4Rmeg and α 4 five types have been found. These regulatory subunits bind with PPP4C to form heterodimers or polymers that map to different suborganelles to perform their respective functions.For example, PP4R1 may reduce the activity of PPP4C or narrow its action range [25]; PP4R2 carries PPP4C to the centrosome and is involved in mitosis [26]; PP4R3 forms heteropolyplex with PP4R2 and PPP4C, which is involved in DNA damage repair process [27]; α 4 may activate the mTOR signaling pathway [8].

In recent years, researchers have shown significant interest in the connection between PPP4C and tumor. Wang et al. [21], in 2008, first tested the expression of PPP4C in human solid tumor samples by immunohistochemistry. The findings indicated a substantial difference in PPP4C expression between benign lesions and breast and lung cancer samples, pointing to a possible link between increased PPP4C expression and the development of breast and lung cancer. A subsequent study by Weng et al. [22] confirmed that the protein and mRNA levels of PPP4C were higher in pancreatic ductal carcinoma samples than in paired adjacent tissues. Furthermore, analysis of PPP4C expression and its clinical pathological characteristics through immunohistochemistry indicated that high PPP4C expression was linked to tumor recurrence, and patients with high PPP4C expression experienced lower DFS or OS than those with low expression. Univariate and multivariate analysis further confirmed that PPP4C serves as an independent risk factor for patient outcome.

In this study, using a pan-cancer sample taken from the TCGA dataset, we first examined the PPP4C mRNA expression level. PPP4C mRNA levels were shown to be significantly higher in various malignancies, including DLBCL, when compared to normal tissues. Two separate GEO datasets provided further confirmation of the differential expression of PPP4C in DLBCL, consistent with previous findings in colorectal cancer, lung, pancreatic ductal adenocarcinoma, and breast cancer. Both indicate a potential role of PPP4C as a cancer-promoting gene. Subsequently, tissue microarray IHC staining of DLBCL tumors revealed increased PPP4C expression in DLBCL patients with ECOG > 2, but no correlation with sex, age, Ann Arbor stage and so on. Crucially, OS and PFS durations were substantially shorter in DLBCL patients with high PPP4C expression than in those with low PPP4C expression. These above findings suggest that PPP4C might be crucial for the growth of DLBCL and tumor metastasis. Furthermore, subgroup analysis indicated that PPP4C is particularly valuable for predicting the prognosis of high-risk DLBCL patients (IPI > 2, ECOG > 2, stage III–IV, and non-GCL). This highlights the potential significance of PPP4C as a prognostic marker specifically for high-risk DLBCL patients. However, to completely understand PPP4C's biological involvement in increasing DLBCL development and the underlying regulatory mechanisms, more research is necessary.

In conclusion, our study showed that PPP4C is substantially expressed in DLBCL tissues and that that there is a significant correlation between high PPP4C expression and a bad prognosis for DLBCL patients. PPP4C may therefore be a helpful predictor of outcome for DLBCL patients.

## Data Availability

The datasets used and/or analyzed during the current study are available from the corresponding author upon reasonable request.
